# Mucopolysaccharidosis type I in 21 Czech and Slovak patients: Mutation analysis suggests a functional importance of C-terminus of the IDUA protein

**DOI:** 10.1002/ajmg.a.32812

**Published:** 2009-04-24

**Authors:** Alzbeta Vazna, Clare Beesley, Linda Berna, Larisa Stolnaja, Helena Myskova, Michaela Bouckova, Hana Vlaskova, Helena Poupetova, Jiri Zeman, Martin Magner, Anna Hlavata, Bryan Winchester, Martin Hrebicek, Lenka Dvorakova

**Affiliations:** 1 Institute of Inherited Metabolic Disorders, First Faculty of Medicine and General Teaching Hospital, Charles University in PraguePrague, Czech Republic; 2 Biochemistry Research Group, UCL Institute of Child Health, University College LondonLondon, UK; 3 Department of Paediatrics and Adolescent Medicine, First Faculty of Medicine and General Teaching Hospital, Charles University in PraguePrague, Czech Republic; 4 2nd Department of Paediatrics, University Children's Hospital, Medical Faculty, Comenius UniversityBratislava, Slovak Republic

**Keywords:** mucopolysaccharidosis I, α-l-iduronidase, mutation, polymorphism, haplotype

## Abstract

Mucopolysaccharidosis type I (MPS I) is an autosomal recessive lysosomal storage disorder that is caused by a deficiency of the enzyme α-l-iduronidase (IDUA). Of the 21 Czech and Slovak patients who have been diagnosed with MPS I in the last 30 years, 16 have a severe clinical presentation (Hurler syndrome), 2 less severe manifestations (Scheie syndrome), and 3 an intermediate severity (Hurler/Scheie phenotype). Mutation analysis was performed in 20 MPS I patients and 39 mutant alleles were identified. There was a high prevalence of the null mutations p.W402X (12 alleles) and p.Q70X (7 alleles) in this cohort. Four of the 13 different mutations were novel: p.V620F (3 alleles), p.W626X (1 allele), c.1727 + 2T > G (1 allele) and c.1918_1927del (2 alleles). The pathogenicity of the novel mutations was verified by transient expression studies in Chinese hamster ovary cells. Seven haplotypes were observed in the patient alleles using 13 intragenic polymorphisms. One of the two haplotypes associated with the mutation p.Q70X was not found in any of the controls. Haplotype analysis showed, that mutations p.Q70X, p.V620F, and p.D315Y probably have more than one ancestor. Missense mutations localized predominantly in the hydrophobic core of the enzyme are associated with the severe phenotype, whereas missense mutations localized to the surface of the enzyme are usually associated with the attenuated phenotypes. Mutations in the 130 C-terminal amino acids lead to clinical manifestations, which indicates a functional importance of the C-terminus of the IDUA protein. © 2009 Wiley-Liss, Inc.

## INTRODUCTION

Mucopolysaccharidosis type I (MPS I) is an autosomal recessive lysosomal storage disorder which is caused by a deficiency of the enzyme α-l-iduronidase (IDUA, EC 3.2.1.76; OMIM *252800). This leads to widespread accumulation of the sulfated glycosaminoglycans (GAGs) dermatan sulfate and heparan sulfate inside lysosomes [Hopwood and Morris, 1990; Neufeld and Muenzer, 2001].

Although there is a continuous spectrum of MPS I clinical phenotypes of varying severity, patients are usually classified into three clinical subtypes (Hurler syndrome, MPS IH, OMIM #607014; Scheie syndrome, MPS IS, OMIM #607016; Hurler/Scheie phenotype, MPS IH/S, OMIM #607015). The subtypes are differentiated by the presence or absence of mental retardation, the severity of visceral manifestation, the age of onset and life span. Clinical diagnosis of all MPS I subtypes is confirmed by elevated levels of dermatan and heparan sulfate in the urine and deficiency of IDUA enzyme activity in leukocytes or fibroblasts [Neufeld and Muenzer, 2001].

Hematopoietic stem cell transplantation is the method of choice for patients with a high risk of mental retardation [Vellodi et al., 1997; Peters et al., 1998; Boelens et al., 2007], while enzyme replacement therapy (ERT) is restricted to the patients with the less severe forms of the disease [Kakkis et al., 2001; Wraith et al., 2004; El Dib and Pastores, 2007]. Genotype analysis of the *IDUA* gene in MPS I families provides prognostic counseling for treatment options and reproductive planning.

To date, over 100 different pathogenic mutations in the *IDUA* gene have been reported in the Human Genome Mutation Database (HGMD; http://www.hgmd.cf.ac.uk/). Moreover, 30 nonpathogenic polymorphisms, seven of them changing an amino acid residue, have been described [Scott et al., 1995; Beesley et al., 2001; Terlato and Cox, 2003].

Despite the high degree of molecular heterogeneity, some mutations show a higher prevalence in certain geographic locations. Among Caucasian patients, two mutant alleles, p.W402X and p.Q70X, are prevalent [Bunge et al., 1994, 1995; Gort et al., 1998], while p.P533R is frequent in Mediterranean patients [Alif et al., 1999; Chkioua et al., 2007]. The mutations p.W402X and p.Q70X are regularly associated with the most severe phenotype and apparently have the highest genotype–phenotype correlation.

The *IDUA* gene has been extensively studied in patients from various nations and ethnicities [Scott et al., 1995; Lee-Chen et al., 1998; Alif et al., 1999; Venturi et al., 2002; Laradi et al., 2005]. With the exception of one article [Voskoboeva et al., 1998], there is no report concerning the *IDUA* gene in the Slavic nations. This report comprises patients from former Czechoslovakia, the Czech Republic, and Slovakia.

## MATERIALS AND METHODS

### Subjects

During the last 30 years, MPS I has been diagnosed in 21 patients from 20 Czech and Slovak families (15 millions inhabitants total). Sixteen patients had the severe form of the disease (MPS IH), two siblings had the less severe form (MPS IS) and three had the intermediate MPS IH/S. The frequency of MPS I, estimated according to the method used by Poorthuis et al. 1999, is 0.7:100,000 in the Czech Republic and 1.32:100,000 in Slovakia [Poupetova et al., unpublished work].

The patient phenotypes were assessed according to the age of onset of clinical symptoms and their progression [Pastores et al., 2007]. The clinical data of the patients enrolled in this study are summarized in Table I. The primary clinical diagnosis was confirmed biochemically both by the demonstration of an increased excretion of urinary dermatan sulfate and heparan sulfate [Dembure and Roesel, 1991] and by a deficiency of IDUA activity in leukocytes of peripheral blood using the artificial substrate 4-methylumbelliferyl α-l-iduronide (Glycosynth Ltd., Warrington, Cheshire, England) [Young, 1992]. There was either no or very low residual enzyme activity in all patient samples, regardless of their phenotype.

**Table I:** Clinical Characterization and Genotypes of the Czech and Slovak Patients With MPS I

Patient number/sex	Phenotype	Age at which clinical signs were first noted/age of dg/Age at review	Short stature/macrocephaly/joint stiffness	Mental Development	Hepatomegaly/splenomegaly	Cardiac disease	Others	Pathogenic variations	Other changes
1/M	H	4m/2y/2y	Yes/yes/yes	DQ 54–62 at 2y	+++/+++	Hypertrophic cardiomyopathy, systolic murmur 3/6	Umbilical hernia, respiratory infections; large tongue; died at preschool age	p.W402X, p.W402X	—
2/M	H	1y/13m/5y	No/no/yes	Normal development till 1y, DQ 79 at 2y	Before HSCT ++/++; after +/+	Systolic murmur 1/6, EF 68% at 4y	Umbilical and inguinal hernia, hearing loss, large tongue, HSCT in 2y (chimerism)	p.W402X, p.W402X	—
3/F	H	6m/13m/14m	No/no/no	DQ 92 in 13m	++/+	Hyperechogenic mitral valve with insufficiency of 1st–2nd grade	HSCT at 16m (performed in March 2008)	p.W402X, p.W402X	—
4/M	H	3m/5m/8m	U/yes; progressive hydrocephalus since 3m/U	Delayed since 3m	++/+	No at 8m	Quadruhyperreflexy at 8m; died at 2y	p.W402X, p.A327P	—
5/M	H	1y/3y/3y	U/U/yes	Delayed since 2nd y	++/++	LV hypertrophy at 3y	Glaucoma	p.W402X, p.V620F	p.R105Q, p.N181, p.A314, p.T410, p.V454I, p.R489
6/M	H	1m/18m/18m	No/no/yes	Delayed since 1y	++/no	LV hypertrophy at 3y	Inguinal bilat. and umbilical hernia, glaucoma	p.W402X, n.i.	p.A8, p.A20, p.N297
7/F	H	8m/10m/5y	No/yes at 8m/yes at 8m	DQ 110 at 1y; DQ 115 at 5y	Before HSCT +++/+; at 5y +/no	No at dg, no at 5y	Umbilical hernia, HSCT at 1y; 4 years after HSCT: normal psychomotor development, hearing problems, strabismus, normal IDUA activity in leucocytes, genua valga; carpal tunnel syndrome	p.W402X, c.1650 + 5G > A (splicing error)	—
8/M	H/S	2,5y/3,5y/16y	No/yes/yes	Borderline DQ at 3y; IQ 97 at 15y	Before HSCT +/+; after HSCT no/no	No at 3y, hemodynamic nonsignificant findings on aortic and mitral valves without progression at 16y	Umbilical hernia, HSCT at 5y, normal IDUA activity at 16y; carpal tunnel syndrome	p.W402X, p.E640Cfs	—
9/F	H/S	3–4y/4y/20y	No/yes/yes, severe and progressive	Uneven development at 2y; IQ 117 at 12y	At dg +++/no; at 12y +/+	Normal at 4y; progressive hypertrophic cardiomyopathy	ERT since 18y; carpal tunnel syndrome; large tongue; died accidentally at 21y	p.W402X, p.E640fs	—
10/M	H	2m/2m/2m	U/no/U	Delayed since early infancy	++/++	U	Umbilical hernia, large tongue; HSCT at 5m; died at 6m due to severe GVHD	p.Q70X, p.Q70X	p.Q33H/Q33H, p.L118/L118
11/M	H	20m/3y/9y	No/yes/yes	Delayed since 2nd y; DQ 73 before HSCT; mental regress after HSCT, DQ 45 at 5y	Before HSCT ++/++; at 9y no/no	No	Umbilical hernia, sensorineural hearing loss; HSCT at 3y; severe acute GVHD, normal IDUA activity	p.Q70X, p.Y167X	p.Q33H/Q33H, p.R105Q, p.N181, p.A314, p.T410, p.V454I, p.R489
12/F	H	5m/5y/5y	Yes/yes/yes	Delayed	+++/++	U	Umbilical hernia, progressive deafness; died at 7y	p.Q70X, p.D315Y	p.A8, p.A20, p.Q33H, p.L118
13/F	H	Infancy/1,5y/5y	U/U/yes	Delayed	Yes	U	Carpal tunnel syndrome	p.Q70X, c.1650 + 5G > A (splicing error)	p.Q33H, p.R105Q, p.N181, p.A314, p.T410, p.V454I, p.R489
14/M	H	24m/3y/8y	Yes/yes/yes	Delayed since infancy	+++/++	Cardiomyopathy, involvement of valves	Inguinal hernia, vision and hearing impairment, ear inflammations, leukodystrophy in MRI, cervical canal stenosis in MRI at 7y, large tongue, tracheostomy; died at 9y	p.Q70X, p.W626X	p.Q33H/Q33H, p.L118
15/F	H/S	Infancy/10m/12y	Yes/yes/yes	Delayed	++/++	Systolic murmur 3–4/6	Inguinal hernia, large tongue; after ERT: decreased illness (obstructive bronchitis), decreased excretion of GAGs in the urine, decreased organomegaly, increased mobility	p.Q70X, p.R628X	p.A8, p.A20, p.Q33H, p.L118
16/M	H	Infancy/2,5y/3y	No/yes; hydrocephalus/U	Delayed since infancy; at 3y DQ 68	++/no	Mild LV hypertrophy	Frequent respiratory infections, large tongue. After ERT: decreased illness (obstructive bronchitis), decreased excretion of GAGs in the urine, decreased organomegaly, increased mobility; died at 5y	p.A327P, c.1727 + 2T > G (splicing error)	p.Q33H
17/F	S	9y/10y/10y	Yes/no/no	VIQ 92; PIQ 84; CIQ 86	++/no	Mild septal hypertrophy; mitral valve dysplasia	Umbilical hernia	p.Q380R, p.Q380R	p.A8/A8, p.A20/A20
18/M	S	15y/15y/15y	Yes/no/yes	At 7y DQ 93; at 15y VIQ 77; CIQ 79	++/+	No	Umbilical/carpal tunnel syndrome	p.Q380R, p.Q380R	p.A8/A8, p.A20/A20
19/F	H	Infancy/3y/17y	Yes/U/yes	Delayed	+++/+++	Systolic murmur 3/6/combined defect of mitral valve	Umbilical hernia, hirsutism, large tongue	p.H539TfsX21, c.1650 + 5G > A (splicing error)	p.A8, p.A20, p.Q33H
20/F	H	Infancy/2y/2y	Yes/U/U	Delayed since early infancy	Yes	Mild LV hypertrophy	Frequent respiratory infections, vision impairment and hearing loss	p.V620F, p.V620F	p.R105Q/R105Q, p.N181/N181, p.A314/A314, p.A361T/A361T, p.T388/T388, p.T410/T410, p.V454I/V454I, p.R489/R489
21/M	H	1m/1m/12m	U/U/no	Delayed since infancy	At dg +/no; at 12m no/no	Mild LV hypertrophy	Inguinal bilat., umbilical hernia	ND	

Patients 17 and 18 are siblings.

+, Mild; ++, moderate; +++, severe.

Coarse facial features present in all patients, very mild in Patient no 7, mild in Patients 17 and 18.

All patients had dysostosis multiplex.

All patients except Patient 18 had corneal clouding.

U, unknown; n.i., not identified; GVHD, graft versus host disease; HSCT, hematopoietic stem cell transplantation; DQ, developmental quotient; VIQ, verbal IQ; PIQ, performance IQ; EF, ejection fraction.

Peripheral white blood cell DNA from 20 patients was screened for *IDUA* gene mutations. This study was approved by an Institutional Review Board of the General University Hospital in Prague and was conducted in accordance with institutional guidelines. DNA from umbilical cord blood samples from 100 Czech anonymous controls was used for the investigation of polymorphisms and haplotypes in the general population.

### Sample Preparation

Genomic DNA and total RNA were extracted from peripheral white blood cells. Genomic DNA was isolated using QIAamp columns (Qiagen GmbH, Hilden, Germany). The method of Chomczynski and Sacchi 1987 with Trizol lysis (Invitrogen, Carlsbad, CA) was used for total RNA extraction [Chomczynski and Sacchi, 1987]. Messenger RNA was reverse-transcribed using SuperScriptII reverse transcriptase (Invitrogen) and oligo dT_18_ according to the manufacturer's instructions.

### IDUA Mutation Analysis

The *IDUA* gene was amplified from genomic DNA in 13 fragments which covered the entire *IDUA* coding region, exon–intron boundaries and some of the 5′- and 3′-untranslated regions. Primers and PCR reaction conditions have been previously described [Beesley et al., 2001].

PCR products were gel-purified, extracted using Wizard® SV Gel and PCR Clean-Up System (Promega, Madison, WI) and directly sequenced on automated fluorescent sequencers Alf Express (Pharmacia, Piscataway, NJ), ABI 3100-Avant (Applied Biosystems, Carlsbad, CA) or MegaBACE capillary DNA sequencer (Amersham Biosciences, Amersham, UK). PCR products containing mutations were re-sequenced in both directions and the mutations were further confirmed by restriction analysis. If the mutation did not alter a restriction enzyme site, one was created artificially by the amplification created restriction site (ACRS) method [Haliassos et al., 1989], specific primers shown in Supplementary Material. Heterozygous deletions were characterized by cloning of the appropriate PCR products into pCR®2.1-TOPO plasmid using the TOPO TA Cloning kit (Invitrogen) and by sequencing of clones containing either of the two alleles.

### IDUA Polymorphism Analysis

Polymorphisms occurring in patients were identified by DNA sequencing. When heterozygous polymorphic markers were found, parental samples were analyzed for haplotype determination. Frequent polymorphisms p.A8, p.A20, p.Q33H, p.R105Q, and p.L118 were examined in control samples. Polymorphisms p.A8, p.A20, and p.Q33H were analyzed by direct sequencing of exon 1, for p.R105Q an ARMS method (see supporting information for details which may be found in the online version of this article) was optimized [Ferrie et al., 1992]. Polymorphism p.L118 was analyzed by PCR/RFLP using *Kpn*I digestion (Takara Bio Inc., Otsu, Shiga, Japan).

### Site-Directed Mutagenesis

The mutations were introduced into the wild-type *IDUA* cDNA, which was cloned into the plasmid vector pSP72 (Promega), using the QuikChange® Site-Directed Mutagenesis Kit (Stratagene, La Jolla, CA) (see supporting information for details which may be found in the online version of this article). Each clone used for the expression study was sequenced to confirm that no other sequence changes had been introduced. The mutant transcript was removed from the pSP72 vector by *Eco*RI digestion, gel purified (Qiagen) and ligated into the mammalian expression vector pIRES2-EGFP (Clontech, Mountain View, CA). Correct orientation of the mutant *IDUA* gene was confirmed by *Kpn*I digestion (NEB, Ipswich, MA).

### Transfection of CHO Cells and Enzyme Assay

Chinese Hamster Ovary (CHO) cells were cultured in Dulbecco's Modified Eagle's Medium (Invitrogen) at 37°C in a 5% CO_2_/air atmosphere. Cells were transfected with either the wild-type or a mutant cDNA construct using Lipofectamine (Invitrogen). For each experiment, 2 × 10^5^ cells were seeded 24 hr prior to transfection. Each construct was transfected in triplicate using 0.4 µg DNA and 5 µl Lipofectamine (Invitrogen). Transfection complexes were removed after 6 hr and replaced with full growth medium. After 24 hr the efficiency of transfection was estimated by the number of cells expressing EGFP, visualized using an inverted fluorescent microscope. Cells were removed and washed twice with phosphate-buffered saline (PBS) and re-suspended in 1 ml PBS. After centrifugation at 2,000 rpm for 10 min, the cells were re-suspended in 50 µl of sterile water and freeze-thawed three times. IDUA enzyme activity was measured as described [Young, 1992], using the artificial substrate 4-methylumbelliferyl alpha-l-iduronide. After incubation for 1 hr at 37°C, the reaction was terminated by the addition of 1.14 ml of 0.25 M glycine NaOH buffer, pH 10.4. The fluorescence of released 4-methylumbelliferone was measured using a Luminescence Spectrometer Model LS50B (Perkin Elmer, Waltham, MA), with an excitation wavelength 365 nm and an emission wavelength of 450 nm. The protein concentration was determined using bicinchoninic acid [Smith et al., 1985].

### Multiple Alignments

**Fig 1:**
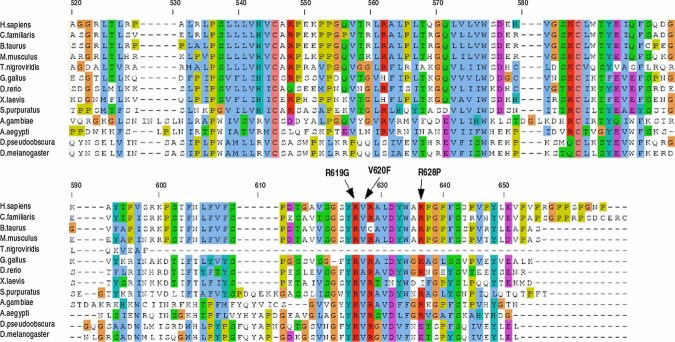
Multiple alignment of proteins homologous to residues 523–653 of the human IDUA protein. The C-terminus of the human IDUA, which does not have a counterpart in many of the proteins from the glycohydrolase family 39, is conserved among multiple species. The residues affected by mutations in MPS I are shown by the arrows. The numbering of residues corresponds to human IDUA. Accession numbers of the sequences used in the alignment: *Homo sapiens*: NP_000194.2, *Canis familiaris*: Q01634, *Bos taurus*: XP_877410.2, *Mus musculus*: NP_032351.1, *Tetraodon nigroviridis*: CAG12584.1, *Gallus gallus*: NP_001026604.1, *Danio rerio*: CAM46905.1, *Xenopus laevis*: AAH77919.1, *Strongylocentrotus purpuratus*: XP_796813.2, *Anopheles gambiae*: XP_314521.3, *Aedes aegypti*: EAT44203.1, *Drosophila pseudoobscura*: XP_001356788.1, *Drosophila melanogaster*: NP_609489.1. [Color figure can be viewed in the online issue, which is available at http://www.interscience.wiley.com.]

**Fig 2:**
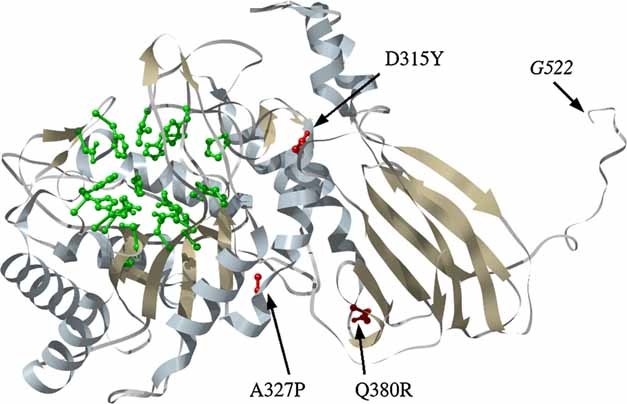
Missense mutations in the 3D structure of the IDUA model of Rempel et al. 2005. The mutated residues are shown in red and the residues depicted in green are predicted to be within the active site. The model does not display the structure of the 130 amino acids at the C terminus (523–653). The last shown residue is G522 (for explanation please see the text). [Color figure can be viewed in the online issue, which is available at http://www.interscience.wiley.com.]

Residues 523–653 of the human IDUA sequence were submitted to a protein BLAST search (http://www.ncbi.nlm.nih.gov/BLAST/) and sequences of proteins with significant homology were retrieved from the databases and aligned using ClustalW. Multiple alignment was visualized using Jalview (Fig. .1) [Clamp et al., 2004]. The positions of missense mutations in the predicted 3D structure of IDUA were examined using UCSF Chimera http://www.cgl.ucsf.edu/chimera [Pettersen et al., 2004], which was also used for the preparation of Figure 2.

## RESULTS

### Identification and Characterization of Mutations

We identified both mutant alleles in 19 patients from 18 families. In Patient 6, only one mutation was detected (Table I). Of the 13 different mutations, 9 have been described previously and 4 were novel (p.V620F, p.W626X, c.1727 + 2T > G and c.1918_1927del). The prevalent Caucasian mutations p.W402X and p.Q70X were also the most frequent among our patients, and represented 47% of the total number of patient alleles (Table II). Four of the patients (Patient 1, 2, 3, and 10) were homozygous for one of these mutations, while only five patients had neither p.W402X nor p.Q70X (Table I). In addition to the prevalent mutations, four other mutant alleles were recurrent, two of them novel (p.V620F and c.1918_1927del).

**Table II:** Characterization of Mutated Alleles in Czech and Slovak MPS I Patients

Location	Nucleotide change^a^	Predicted effect on protein	Codon change	Frequency^b^	Restriction enzyme^a^	Refs.
Exon 2	c.208C > T	p.Q70X	CAG > TAG	7 (17%)	*Bfa*I/*Fsp*BI	Scott et al. 1992b
Exon 5	c.501C > A	p.Y167X	TAC > TAA	1 (3%)	*Rsa*I	Beesley et al. 2001
Exon 7	c.943G > T	p.D315Y	GAC > TAC	1 (3%)	*Rsa*I	Scott et al. 1995, Li et al. 2002
Exon 8	c.979G > C	p.A327P	GCG > CCG	2 (5%)	*Bst*UI	Bunge et al. 1994
Exon 8	c.1139A > G	p.Q380R	CAG > CGG	4 (10%)	*Pvu*II	Scott et al. 1995
Exon 9	c.1205G > A	p.W402X	TGG > TAG	12 (30%)	*Bfa*I/*Fsp*BI	Scott et al. 1992a
Exon 11	c.1614delG	p.H539TfsX21		1 (3%)	*Apa*LI	Scott et al. 1993a
Intron 11	c.1650 + 5G > A (IVS11 + 5G > A)	Splicing error		3 (7%)	*Nde*I (ACRS)	Venturi et al. 2002
Intron 12	**c.1727 + 2T > G** (IVS12 + 2T > G)	Splicing error p.C577GfsX15		1 (3%)		Novel
Exon 14	**c.1858G > T**	p.V620F	GTT > TTT	3 (7%)	*Tsp*509I (ACRS)	Novel
Exon 14	**c.1877G > A**	p.W626X	TGG > TAG	1 (3%)	*Bfa*I	Novel
Exon 14	c.1882C > T	p.R628X	CGA > TGA	1 (3%)	*Apa*I	Beesley et al. 2001
Exon 14	**c.1918_1927del**	p.E640Cfs	del.GAGGTCCCTG	2 (5%)		Novel

a Novel variants in boldface type.

b The total number of alleles N = 40 used for frequency estimation includes also unspecified mutated allele.

c ACRS, amplification created restriction site.

The mutation c.1727 + 2T > G was further analyzed on the transcript level. The insertion of four intronic nucleotides resulted in the shift of the splice donor site of intron 12 (r.1727_1728ins1727 + 1_1727 + 4), presumably leading to a frameshift, which started at 577th amino acid residue and ended at a premature stop codon 15 amino acid residues downstream (p.C577GfsX15).

Three novel mutations (p.V620F, p.W626X, and c.1918_1927del) and a previously described mutation, p.D315Y, were analyzed further by expression studies. The null allele p.W402X was used as a positive control. The results are summarized in Table III and show that the mutations p.D315Y, p.V620F and p.W626X lead to undetectable enzyme activity, whereas the deletion of 10 bases in the last exon (c.1918_1927del) produced measurable residual enzyme activity.

**Table III:** α-l-Iduronidase Activity in CHO Cells Transiently Transfected With Wild-Type (wt) or Mutagenized cDNAs

Transfection vector	α-l-iduronidase activity (nmol/h/mg cell protein)	Associated phenotype
CHO	4.1	
pIRES2	4.7	
pIRES2/wt	1005.7	
pIRES2/W402X (control null mutation)	6.1	IH
pIRES2/D315Y	4.2	IH
pIRES2/V620F	8.2	IH
pIRES2/W626X	4.6	IH
pIRES2/E640Cfs	29.1	IH/S

Values are the mean of 6 experiments.

### Analysis of Polymorphisms and Haplotypes

By analyzing the entire *IDUA* coding sequence, we identified 13 previously described polymorphisms (Table I). The analysis of SNPs in patient and parental samples enabled us to derive haplotypes in 34 of the mutant alleles. The haplotypes are determined mainly by five SNPs located in exons 1–3 (p.A8, p.A20, p.Q33H, p.R105Q, and p.L118), while eight remaining SNPs of exons 5–10 (p.N181, p.N297, p.A314, p.A361T, p.T388, p.T410, p.V454I, p.R489) show little heterogeneity with a high prevalence of T-C-G-G-G-C-G-C nucleotides.

Although the haplotype for p.V620F could not be determined in Patient 5, it differs from the p.V620F haplotype of Patient 20 in polymorphisms p.A361T and p.T388 (Table I).

The polymorphisms p.A8, p.A20, p.Q33H, p.R105Q, and p.L118, which were found to be frequent in patients, were examined in 200 control alleles. A substantial heterogeneity was found with the frequencies of polymorphic alleles of 43%, 45%, 21%, 13%, and 26%, respectively.

From the total number of 100 control samples, 28 were homozygous for the studied polymorphic markers (p.A8, p.A20, p.Q33H, p.R105Q, and p.L118) and 2 were heterozygous in one marker. Therefore, 30 samples (60 alleles) could be used to define haplotypes occurring in the Czech population (Table IV). In patients, haplotypes based on the 5 polymorphisms could be determined in 34 alleles.

**Table IV:** Haplotypes in Mutated and Control Alleles (Based on 5 Exonic Polymorphisms)

Haplotype	A8	A20	Q33H	R105Q	L118	Frequency in control alleles	Frequency in mutated alleles	Association of haplotype with mutation (Patient no)
I	A	A	G	G	C	27 (45%)	7 (20%)	p.D315Y (12), p.Q380R (17,18), p.R628X (15), allele with n.i. mutation (6)
II	C	G	G	G	C	13 (22%)	17 (50%)	p.W402X (1,2,3,4,6,7,8,9), c.1650 + 5G > A (7,13), p.A327P (4,16), c.1918_1927del (8,9)
III	C	G	T	G	T	9 (15%)	4 (12%)	p.Q70X (10,12,15)
IV	C	G	T	G	C	3 (5%)	2 (6%)	p.Y167X (11), c.1727 + 2T > G (16)
V	C	G	G	G	T	4 (7%)	0	—
VI	A	A	T	G	C	1 (1%)	0	—
VII	C	G	G	A	C	3 (5%)	2 (6%)	p.V620F (20)
VIII	C	G	T	A	C	0	2 (6%)	p.Q70X (11,13)

Haplotypes of mutated alleles were determined by analysis of patient and parental samples. Control alleles heterozygous at most in one polymorphic marker were used.

Total number of control alleles = 60.

Total number of mutated alleles = 34.

Table IV indicates that the two most abundant haplotypes (I and II) show reverse frequencies in the control and patient group, respectively. The haplotypes V and VI were found only in control alleles while the haplotype VIII was linked only with two p.Q70X alleles.

## DISCUSSION

### Mutation Analysis

The full genotype of 19 MPS I patients (including 2 siblings) from 19 Czech and Slovak families has been established, while only 1 mutant allele was found in 1 patient (Patient 6). The second mutation of this patient was not identified but it could be located deep within the intron affecting splicing or in the promoter region affecting transcription.

Nine previously known and four novel mutations were found in our patients. Mutation analysis confirmed the prevalence of two mutations, p.W402X and p.Q70X, which have been found in several different patient groups of European origin [Scott et al., 1995; Gort et al., 1998; Beesley et al., 2001; Li et al., 2002]. In our cohort of patients, the p.W402X and p.Q70X alleles represented 30% and 17%, respectively, which is similar to the frequencies in German and Dutch patients [Scott et al., 1995].

The deleterious effects of three novel and one previously described mutation (p.V620F, p.W626X, c.1918_1927del, and p.D315Y) were confirmed by functional assay. The fourth novel mutation, c.1727 + 2T > G, was proved to be a donor splice site mutation presumably leading to a frameshift and premature termination 15 amino acid residues downstream from the first affected amino acid. The analogous mutation c.1727 + 2T > A has been described previously [Yogalingam et al., 2004]. Therefore, it is highly probable that the novel mutation c.1727 + 2T > G is pathogenic.

### Polymorphisms and Haplotypes

Besides the pathogenic mutations, 13 previously described nonpathogenic sequence variants were identified in this cohort. All of them were exonic and four changed an amino acid residue. SNPs changing the amino acid sequence are of particular interest because it has been suggested that they may modulate the phenotype by affecting IDUA protein stability and/or its catalytic activity [Yogalingam et al., 2004]. According to the literature, the polymorphism p.A361T is suggested to potentiate the deleterious effect of the p.R89Q mutation [Scott et al., 1993b]. In our patient cohort, the polymorphism p.A361T was identified only in Patient 20, who is homozygous for the p.V620F mutation as well as the p.A361T polymorphism. The substitution p.V620F was found to be a severe mutation, which corresponds well to the Hurler phenotype of the patient. However, the potentiating effect of p.A361T cannot be excluded.

The prevalent mutation p.W402X has been reported in association with three different haplotypes [Scott et al., 1995], while only one was found in our group of patients. The same haplotype was found also in association with the novel mutation c.1918_1927del. On the other hand, the second prevalent mutation p.Q70X and a novel mutation p.V620F were associated with two different haplotypes. Moreover, the haplotype associated with p.D315Y in Patient 12 is different from that which can be deduced from the published data [Li et al., 2002]. These findings enlarge the list of mutations associated with more than one haplotype as reviewed in Scott et al. 1995 and show that there is an increasing number of *IDUA* gene mutations that might have more than one origin.

### Genotype–Phenotype Correlation

The correlation between genotype and phenotype is not straightforward in MPS I patients even though there is remarkable intrafamilial clinical presentation. It is generally accepted that the combination of two null alleles usually leads to the severe Hurler phenotype, while the less severe Hurler/Scheie and Scheie forms of the disease are associated with at least one mild allele that produces some residual enzyme activity [Scott et al., 1995; Beesley et al., 2001; Terlato and Cox, 2003]. The resulting phenotype may be modified by genetic background including functional polymorphisms and haplotypes, and environmental factors. Genetic background may influence various intracellular processes, like nonsense mediated decay or alternative splicing effects [Scott et al., 1993b, 1995; Yogalingam et al., 2004].

Although the clinical spectrum in MPS I patients is continuous and the phenotypic determination is influenced by the effect of therapy in some of our patients, a genotype–phenotype correlation may be observed in several cases. Patients 1, 2, 3, 10, 11, and 14 who carry nonsense mutations on both alleles presented with the severe phenotype. These findings are in accordance with the previously published data [Scott et al., 1995; Beesley et al., 2001; Terlato and Cox, 2003]. However, recently a patient homozygous for p.W402X presenting with the less severe Scheie phenotype was described [Pereira et al., 2008]. Unfortunately, no additional data concerning the patient's *IDUA* gene characterization were published.

We observed discrepancy between genotype and phenotype in Hurler/Scheie Patient 15, who has two nonsense mutations, p.Q70X/p.R628X (the compound heterozygosity was confirmed by analysis of parental samples). Haplotype 4 associated with p.R628X was found in patients with different phenotypes (Patients 12, 15, 17, 18) and does not seem to influence the clinical features significantly. Three patients homozygous for p.R628X have been described in the literature; two of them had the Hurler phenotype [Beesley et al., 2001] and one had the MPS IH/S phenotype [Venturi et al., 2002]. The slightly milder phenotype observed in some patients may be related to the fact that the TGA codon created by the mutation, p.R628X, is a stop codon that has the highest natural read-through potential in comparison with the other two TAA and TAG termination codons [Hein et al., 2004].

In our study, mutation p.Q380R was found homozygously in two siblings with the Scheie phenotype (Patients 17, 18), whilst the same mutation in combination with p.R621X was found in a Hurler/Scheie patient [Beesley et al., 2001]. This suggests that p.Q380R may lead to the production of an enzyme with a residual activity.

Patients 4 and 7 from our cohort had the same combination of mutations and a similar phenotype to two patients previously published by Bunge et al. 1994 and Venturi et al. 2002, respectively.

Patients 8 and 9 who have the MPS IH/S phenotype are compound heterozygotes for p.W402X and the novel deletion (c.1918_1927del). The deletion is predicted to result in a frameshift, which abolishes the natural stop codon, but does not create a new one. Hence, the transcript is predicted to extend towards the 3′ end and an elongated protein will be synthesized. Although deletions are usually associated with a severe phenotype [Terlato and Cox, 2003], the deletion of 10 nucleotides in near proximity to the natural termination of translation appears to permit some functional enzyme to be produced (a residual enzyme activity, ∼3% of normal, was detected). This would explain the milder phenotypes of both patients.

Rempel et al. 2005 constructed a homology model of the IDUA protein on the basis of the crystal structure of another glycosyl hydrolase family 39 protein, beta-xylosidase from *Thermoanaerobacterium saccharolyticum* (E.C. 3.2.1.37). This model showed that missense mutations associated with the severe MPS I phenotypes were localized predominantly in the active site or in the hydrophobic core of the protein model, whilst the majority of missense mutations localized to the surface lead to the attenuated phenotype. The previously described mutations p.A327P and p.D315Y located in the core were found in Hurler patients 4, 12, and 16. Mutation p.Q380R which can be considered a less severe mutation [this study and Beesley et al., 2001] is situated on the surface of the protein (Fig. .2).

The model does not predict the 3D structure of the C-terminus of the protein since the residues 523–653 of the human IDUA enzyme do not have a counterpart in the sequence of xylosidase. The function of the C-terminus is not known, although, as pointed out by Rempel et al. 2005, its similarity to some fibronectin III domains may suggest that it plays a role in protein–protein interactions. Whatever its function, the 130 C-terminal amino acids appear to be important. This suggestion is supported by the identification of causative mutations in this region [Beesley et al., 2001; Lee-Chen et al., 2002]. Even missense mutations, such as the p.V620F identified in this study and p.R619G, p.R628P [Lee-Chen et al., 1999; Matte et al., 2003], are associated with the severe phenotype. This region is conserved among mammalian IDUAs and to a lesser degree also in orthologous proteins from *Danio rerio*, *Drosophila*, *Anopheles*, and *Tetraodon* (Fig. 1). Residues affected by the above missense mutations are highly conserved among multiple species.

To highlight some of the findings, our data show that (1) mutations p.Q70X, p.V620F and p.D315Y might have arisen more that once, (2) the genotype correlated well with the phenotype in the majority, but not in all of the described patients, and (3) the C-terminus, which has no counterpart in the sequence of IDUA orthologs, is apparently important for IDUA enzyme function in humans.

*Note*: After submission of the manuscript, two other MPS IH patients were diagnosed. They were homozygous for p.Q70X and p.W402X, respectively.
