# Supplementary material for: Mucopolysaccharidosis type I in 21 Czech and Slovak patients: Mutation analysis suggests a functional importance of C-terminus of the IDUA protein
Source: Am J Med Genet A. 2009 Apr 24;149A(5):965–74. doi: 10.1002/ajmg.a.32812 (PMC3526155; doi:10.1002/ajmg.a.32812)
Supplement: Supplementary file 1 [file ajmg0149A-0965-SD1.pdf]

Supplemental material

Primers for the ARMS and ACRS PCR and for Quikchange site-directed mutagenesis

**SNP/mutation**	**Sense primer[a]**	**Method**
p.R105Q	5’-ACCGTCCTTCTGCAGGGGGTCCAGTGGCC**G**-3’<br>5’-ACCGTCCTTCTGCAGGGGGTCCAGTGGCC**A**-3’<br>5’-AGCCCTGAATGGACATCCAAGGACTCAGAG-3’	ARMS
p.V620F	5’-AGACACAGGTGCTGTCTCTGGCTCCTACC**A**-3’<br>5’-TATATTGCAAAGGGGGTGATG-3’	ACRS
c.1650+5G>A	5’-GCGCGCCCCGAGAAGCCGCCCGGGCAGGCA**T**-3’<br>5’-GTGACCGCATGGGTGAAG-3’	ACRS
p.D315Y	5’-CCGTGGAGGGCG**T**ACGTGACCTACG-3’<br>5’-CGTAGGTCACGT**A**CGCCCTCCACGG-3’	SDM
p.W402X	5’-GGAGCAGCTCT**A**GGCCGAAGTGTCG-3’<br>5’-CGACACTTCGGCC**T**AGAGCTGCTCC-3’	SDM
p.V620F	5’-GGCTCCTACCGA**T**TTCGAGCCCTGG-3’<br>5’- CCAGGGCTCGAA**A**TCGGTAGGAGCC-3’	SDM
p.W626X	5’-CCTGGACTACT**A**GGCCCGACCAGG-3’<br>5’- CCTGGTCGGGCC**T**AGTAGTCCAGG-3’	SDM
c.1918_1927del	5’-GCAGTGCAGCCCGAAG-3’<br>5’- ATATTGCAAAGGGGGTGA-3’	SDM

[a]Change of sequence in comparison with reference sequence in boldface type.
ARMS, amplification refractory mutation system; ACRS,amplification created restriction site; SDM, site-directed mutagenesis.
